# *Streptococcus pneumoniae *synergizes with nontypeable *Haemophilus influenzae *to induce inflammation via upregulating TLR2

**DOI:** 10.1186/1471-2172-9-40

**Published:** 2008-07-29

**Authors:** Jae Hyang Lim, Unhwan Ha, Akihiro Sakai, Chang-Hoon Woo, Soo-Mi Kweon, Haidong Xu, Jian-Dong Li

**Affiliations:** 1 Department of Microbiology and Immunology, University of Rochester Medical Center, Rochester, New York, NY 14642, USA; 2 Department of Molecular Cell biology, House Ear Institute, Los Angeles, CA 90057, USA; 3 Cardiovascular Research Institute, University of Rochester Medical Center, Rochester, New York, NY 14642, USA

## Abstract

**Background:**

Toll-like receptor 2 (TLR2) plays a critical role in mediating inflammatory/immune responses against bacterial pathogens in lung. *Streptococcus pneumoniae (S. pneumoniae) *and nontypeable *Haemophilus influenzae *(NTHi) were previously reported to synergize with each other to induce inflammatory responses. Despite the relatively known intracellular signaling pathways involved in the synergistic induction of inflammation, it is still unclear if both bacterial pathogens also synergistically induce expression of surface TLR2.

**Results:**

Here we provide direct evidence that *S. pneumoniae *synergizes with NTHi to upregulate TLR2 expression in lung and middle ear of the mice. Pneumolysin (PLY) appears to be the major virulence factor involved in this synergism. Moreover, *S. pneumoniae *PLY induces TLR2 expression via a TLR4-MyD88-NF-κB-dependent signaling pathway. Interestingly, tumor suppressor CYLD acts as a negative regulator of *S. pneumoniae*-induced TLR2 up-regulation via negative-crosstalk with NF-κB signaling.

**Conclusion:**

Our study thus provides novel insights into the regulation of TLR2 expression in mixed bacterial infections.

## Background

Gram-negative bacterium nontypeable *Haemophilus influenzae *(NTHi) and gram-positive bacterium *Streptococcus pneumoniae *(*S. pneumoniae*) are important bacterial pathogens causing lung infections [1,2]. In children, they cause otitis media (OM), the most common childhood infection and the leading cause of conductive hearing loss [3], while in adults, they exacerbate chronic obstructive pulmonary diseases (COPD), the fourth leading cause of death in the United States [1,2]. Although a majority of OM or COPD is mainly associated with a single bacterial pathogen, there is a growing body of evidence that a portion of patients diagnosed with OM or COPD have mixed infections of NTHi and *S. pneumoniae *[4,5]. In the mixed infection of NTHi and *S. pneumoniae*, two bacterial pathogens synergistically induce inflammation through activation of specific host signaling pathway [6,7], which is initiated through the recognition of microbial pathogens by means of cell surface receptors.

Toll-like receptors (TLRs) are a class of pathogen recognition receptors that mediate recognition of pathogen-associated molecular pattern. Among 13 mammalian TLRs, TLR2 plays especially a critical role due to its capability of detecting the widest repertoire of pathogen-associated molecular patterns from a large variety of pathogens, including gram-positive or gram-negative bacteria, mycobacteria, fungi, viruses, and parasites [8].

We previously reported that TLR2 is a key receptor recognizing NTHi components [9] and is highly inducible by multiple pro-inflammatory stimuli including NTHi itself [9-12]. Given the fact that *S. pneumoniae *co-exists with NTHi in lung and middle ear infections and synergistically enhances NTHi-induced inflammation [6], it is of particular interest to investigate the molecular mechanism by which TLR2 is likely induced by NTHi and *S. pneumoniae *in a synergistic manner. In the present study, we showed that *S. pneumoniae *induces TLR2 up-regulation via a cytoplasmic pneumolysin (PLY) through a TLR4-MyD88-NF-κB-dependent signaling pathway. Moreover, *S. pneumoniae *induces expression of tumor suppressor cylindromoatosis (CYLD), which in turn negatively regulates *S. pneumoniae*-induced NF-κB activation and TLR2 expression. These studies thus bring new insights into synergistic regulation of host TLRs in mixed bacterial infections.

## Results

### *S. pneumoniae *pneumolysin induces TLR2 expression in the epithelium of lung and middle ear *in vitro *and *in vivo*

**Figure 1:**
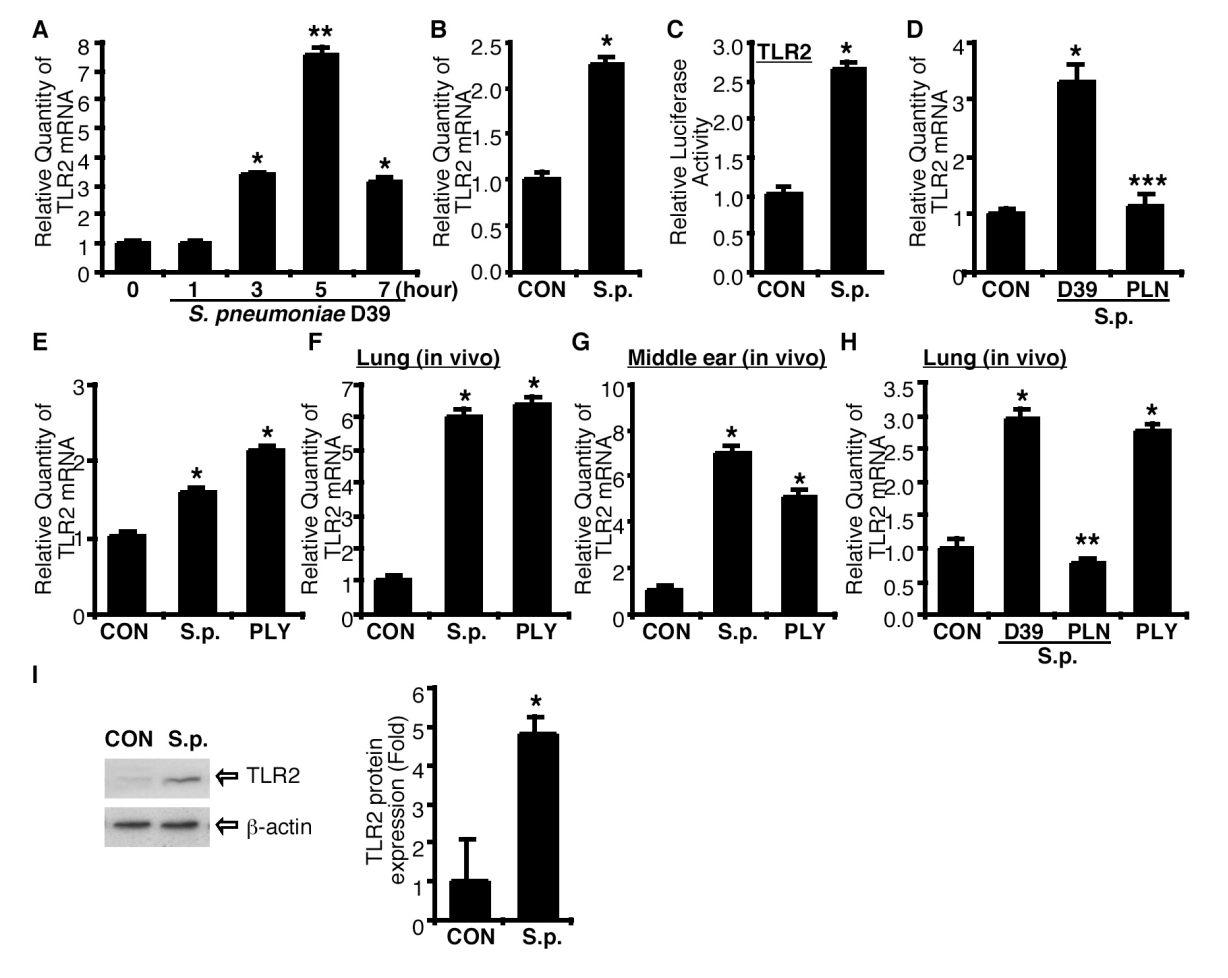
***S. pneumoniae *induces TLR2 up-regulation via pneumolysin in the epithelium of lung and middle ear**. (A) Time dependence of TLR2 up-regulation in response to *S. pneumoniae *(*S.p.*). HeLa cells were treated with *S.p. *for 1, 3, 5 and 7 hours, and relative quantity of TLR2 mRNA expression was measured by Q-PCR analysis. (B)*S.p*.-induced TLR2 mRNA expression in lung epithelial cell A549. Cells were treated with *S.p.*, and relative quantity of TLR2 mRNA expression was measured by Q-PCR analysis 5 hours after treatment. (C) *S.p.*-induced TLR2 promoter activity. HeLa cells were transiently transfected with TLR2 luciferase reporter gene, treated with *S.p.*, and relative luciferase activity was measured 5 hours after treatment. (D) The effects of *S.p. *WT strain D39 and PLY-deficient strain PLN on *S.p.*-induced TLR2 expression. Cells were treated either with *S.p. *D39 or PLN, and mRNA expression of TLR2 was measured by Q-PCR analysis 3 hours after treatment. (E) *S.p.*- and PLY-induced TLR2 mRNA expression in middle ear cell HMEEC-1. Cells were treated with *S.p. *or PLY, and TLR2 mRNA expression was measured 5 hours after treatment by Q-PCR analysis. (F & G) *S.p.*- and PLY-induced TLR2 expressions in the lung and middle ear of the mice. Wild-type mice were intratracheally inoculated with *S.p. *or PLY for *S.p.*-induced pneumonia (F) and inoculated into the middle ear through tympanic membrane for *S.p.*-induced otitis media model (G). Relative quantity of TLR2 mRNA was measured from the lungs of inoculated mice 3 hours after inoculation and from the middle ears of inoculated mice 9 hours after inoculation. (H) The effect of PLY on *S.p.*-induced TLR2 expression was measured using *S.p. *WT strain D39 and PLY-deficient strain PLN in mice *in vivo*. WT mice were intratracheally inoculated with *S.p. *D39 or PLN, or purified PLY, and TLR2 mRNA expression was measured from the lungs of inoculated mice 9 hours after inoculation by Q-PCR analysis. (I) Protein expression of TLR2 is up-regulated following *S.p. *infection. TLR2 protein expression was measured from the lungs WT mice inoculated with *S.p. *by western blotting analysis 6 hours after inoculation (left panel), and band data was analyzed using Kodak Image analysis system and data was presented as Fold induction (right panel). Data are means ± S.D. (n = 3). *, *p *< 0.05 compared with CON, **, *p *< 0.01 compared with CON, ***, *p *< 0.05 compared with *S.p. *D39, CON, control.

We initially investigated the effect of *S. pneumoniae *on TLR2 expression in epithelial cells. As shown in Fig. 1A, TLR2 mRNA expression was induced by *S. pneumoniae *treatment in human epithelial HeLa cells in a time-dependent manner. TLR2 expression was markedly up-regulated at 3 h after treatment, peaked at 5 h and declined thereafter. TLR2 up-regulation by *S. pneumoniae *was also confirmed in human respiratory epithelial A549 cells (Fig. 1B). We next determined if TLR2 up-regulation by *S. pneumoniae *occurs at the transcriptional level. As shown in Fig. 1C, transcriptional activity of TLR2 promoter reporter was also up-regulated by *S. pneumoniae *in epithelial cells. Because pneumolysin (PLY) plays a key role in the pathogenesis of *S. pneumoniae*, we investigated whether PLY is a crucial virulence factor for the TLR2 up-regulation by *S. pneumoniae*. Human respiratory epithelial cells were treated with either *S. pneumoniae *WT D39 or pneumolysin-deficient mutant PLN, and mRNA expression level of TLR2 was then measured by Q-PCR analysis. As shown in Fig. 1D, inoculation of *S. pneumoniae *WT D39 induced TLR2 mRNA expression, whereas *S. pneumoniae *PLN did not induce it. Moreover, purified PLY induced TLR2 expression to a similar extent to *S. pneumoniae *in middle ear HMEEC-1 epithelial cells (Fig. 1E). To further confirm if *S. pneumoniae *also induces TLR2 expression *in vivo*, we examined *S. pneumoniae*- and PLY-induced TLR2 expression *in vivo *using *S. pneumoniae*-induced pneumonia and OM model in mice. As shown in Fig. 1F &1G, *S. pneumoniae *induced TLR2 mRNA expression in both lung and middle ear tissues. Similar results were also observed in PLY-inoculated mice (Fig. 1H). Consistent with our *in vitro *findings, TLR2 expression was up-regulated by *S. pneumoniae *WT D39 and PLY inoculation but not mutant PLN (Fig. 1H). Furthermore, Western blot analysis confirmed TLR2 up-regulation by *S. pneumoniae *at the protein level after intratracheal inoculation of *S. pneumoniae *in WT mice (Fig. 1I). Taken together, our data showed that *S. pneumoniae *PLY induces TLR2 up-regulation in lung and middle ear *in vitro *and *in vivo*.

### *S. pneumoniae *induces TLR2 expression through TLR4-MyD88-NF-κB-dependent signaling pathway

**Figure 2:**
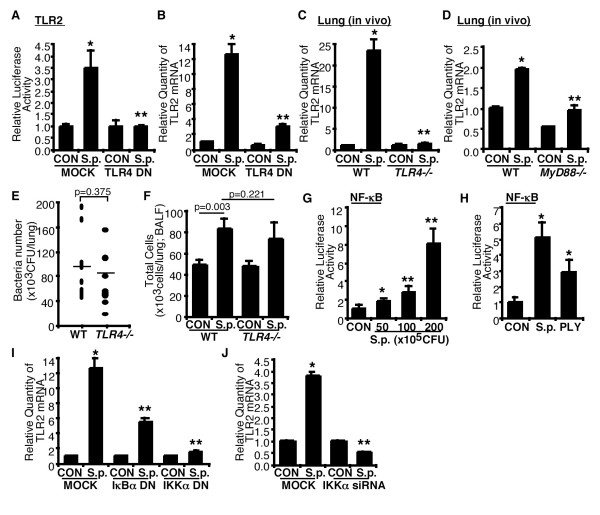
**TLR4-MyD88-NF-κB signaling pathway mediates *S. pneumoniae*-induced TLR2 up-regulation**. (A) Cells were transiently transfected with TLR2 promoter reporter gene with TLR4 DN mutant plasmid or empty vector, and *S.p.*-induced luciferase activity was measured 5 hours after *S.p. *treatment. (B) Cells were transiently transfected either with TLR4 DN mutant plasmid or empty vector, and *S.p.*-induced TLR2 mRNA expression was measured by Q-PCR analysis. (C) WT and TLR4 KO mice were intratracheally inoculated with *S.p.*, and TLR2 mRNA expression was measured from the lungs of inoculated mice by Q-PCR analysis. (D) WT and MyD88 KO mice were intratracheally inoculated with *S.p.*, and TLR2 mRNA expression was measured from the lungs of inoculated mice by Q-PCR analysis. (E) Bacterial number in the lungs of WT and TLR4 KO mice following *S.p. *inoculation. WT and TLR4 KO mice were intratracheally inoculated with 1 × 10^7 ^CFU of *S.p. *D39, and CFU of the lungs were measured 24 hours after inoculation. (F) Inflammatory cell migration into airway in WT and TLR4 KO mice following *S.p. *inoculation. WT and TLR4 KO mice were intratracheally inoculated with 1 × 10^7 ^CFU of *S.p.*, and total cells were measured from the BAL fluid of the lungs of saline- or *S.p.*-inoculated mice 6 hours after inoculation. (G & H) Cells were transfected with NF-κB reporter gene, and *S.p.*- or PLY-induced NF-κB promoter activity was measured as relative luciferase activity. (I & J) Cells were transiently transfected with IκBα DN, IKKα DN, IKKαsiRNA, empty vector, or control siRNA, and *S.p.*-induced TLR2 mRNA expression was measured by Q-PCR analysis. Data in A-D & G-J are means ± S.D. (n = 3). *, *p *< 0.05 compared with CON, **, *p *< 0.05 compared with *S.p. *in MOCK or WT mice, CON, control inoculated with vehicle.

TLRs play a critical role in recognizing extracellular pathogens and transducing the cell surface interaction between microbes and cells to intracellular components. It was previously reported that cytolysins including *Bacillus anthracis *anthrolysin O and *S. pneumoniae *PLY act as TLR4 agonists [13]. Moreover, we reported that PLY triggers TLR4-dependent intracellular signaling pathway in severe *S. pneumoniae *infection in lung [14,15]. Thus, we determined whether *S. pneumoniae *induced-TLR2 up-regulation is mediated by TLR4 *in vitro *and *in vivo*. Perturbing TLR4 signaling using TLR4 DN mutant plasmid inhibited both *S. pneumoniae*-induced TLR2 promoter activity (Fig. 2A) and TLR2 mRNA expression (Fig. 2B) *in vitro*. To further investigate whether TLR4 is also critical for *S. pneumoniae*-induced TLR2 up-regulation *in vivo*, WT and TLR4 KO mice were intratracheally inoculated with *S. pneumoniae*, and TLR2 mRNA expression was measured in the lungs of inoculated mice. As shown in Fig. 2C, *S. pneumoniae *induced TLR2 expression in the lungs of WT mice but not TLR4 KO mice *in vivo*. Because MyD88 is a well-known signaling adaptor for TLR4, we determined whether MyD88 is required for the *S. pneumoniae*-induced TLR2 expression *in vivo*. As shown in Fig. 2D, *S, pneumoniae*-induced TLR2 up-regulation was significantly reduced in MyD88 KO mice compared to WT mice. Having demonstrated a marked decrease in TLR2 expression in TLR4 KO mice, we determined whether TLR4-deficiency caused this decrease via changing bacterial clearance in the lung tissues or inflammatory cell migration into airways. We therefore measured bacteria number from the lungs of *S. pneumoniae*-inoculated WT and TLR4 KO mice 24 hrs after inoculation and also measured inflammatory cell migration into airway from the BAL fluid of vehicle- or *S. pneumoniae*-inoculated WT and TLR4 KO mice 6 hrs after inoculation (Fig. 2E &2F). Neither bacteria number of the lungs nor inflammatory cell migration into airways in TLR4 KO mice was significantly different from those in WT mice. The results reported herein demonstrate that, under the conditions reported here, TLR4-deficiency does not affect the bacterial clearance of *S. pneumoniae *and inflammatory cell migration response to *S. pneumoniae*, and decrease in *S. pneumoniae*-induced TLR2 expression in TLR4 KO mice is independent of both bacteria number and inflammatory cells in the lung *in vivo*. Collectively, our data suggest that *S. pneumoniae *induces TLR2 up-regulation via TLR4-MyD88 signaling pathway.

Because NF-κB plays an important role in mediating PLY-induced host responses, we next determined if NF-κB is also involved in mediating TLR2 up-regulation by PLY [9,10,16]. As shown in Fig. 2G &2H, *S. pneumoniae *induced NF-κB activation in a dose-dependent manner. Similar result was also observed in PLY-treated cells. We further determined the involvement of IKKα-IκBα, the key signaling pathway upstream of NF-κB, in TLR2 induction [6,14]. As shown in Fig. 2I, perturbing IKKα and IκBα signaling by overexpressing DN mutant plasmid inhibited *S. pneumoniae*-induced TLR2 mRNA up-regulation. This finding was further confirmed by using IKKαsiRNA (Fig. 2J). Thus it is clear that *S. pneumoniae*-induced TLR2 up-regulation is mediated by IKKα and IκBα-dependent NF-κB activation.

### *S. pneumoniae *induced-TLR2 up-regulation is negatively regulated by tumor suppressor CYLD

**Figure 3:**
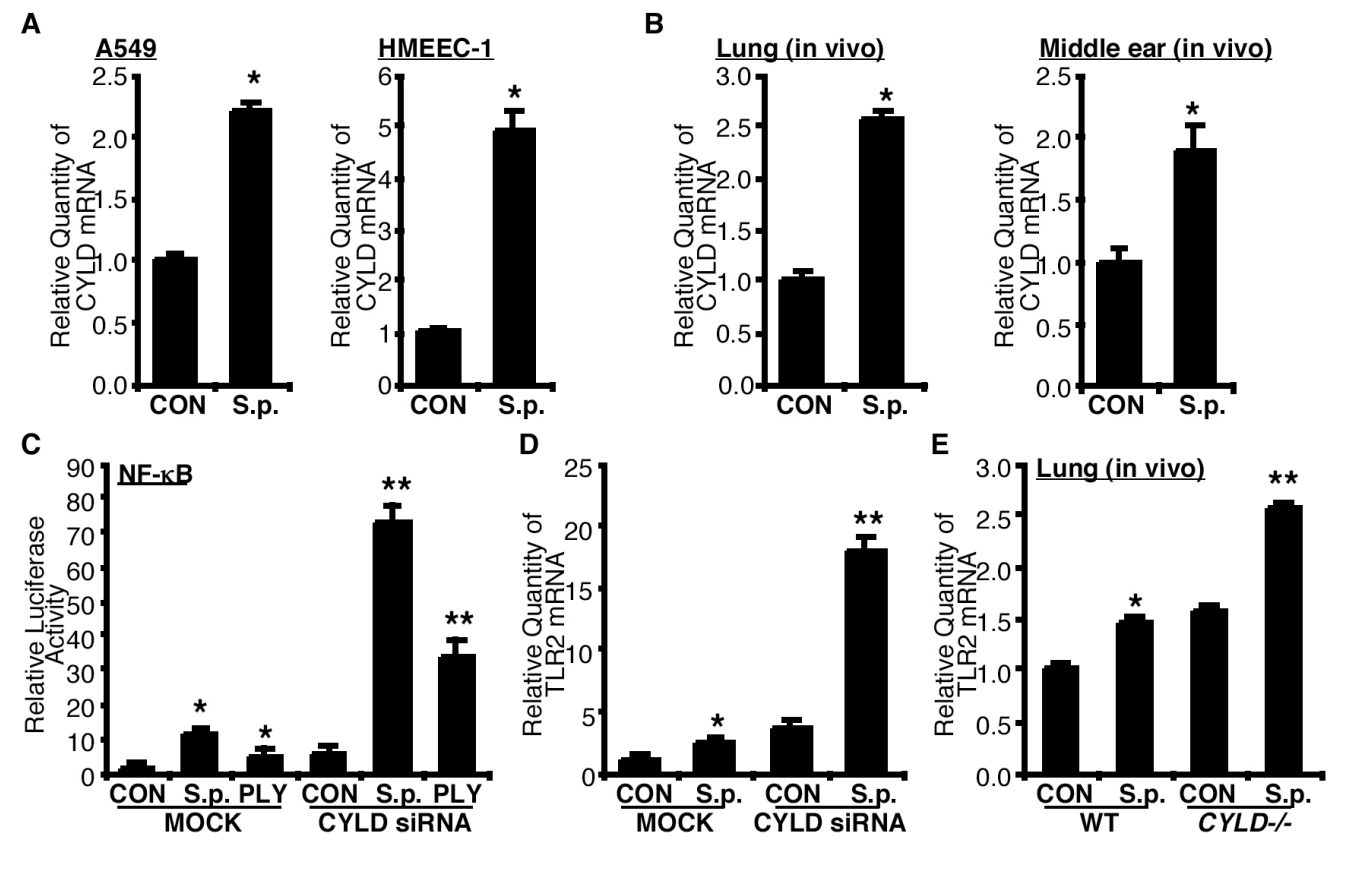
**CYLD negatively regulates *S. pneumoniae*-induced TLR2 up-regulation**. (A) Human alveolar epithelial cell A549 and human middle ear epithelial cell HMEEC-1 were treated with *S.p.*, and CYLD mRNA expression was measured 5 hours after treatment by Q-PCR analysis. (B) WT mice were intratracheally or transtympanically inoculated with *S.p. *for pneumonia and otitis media model, respectively, and CYLD mRNA expression was measured from the lungs and middle ear tissues of inoculated mice 3 hours after inoculation and 9 hours after inoculation, respectively. Data in A & B are means ± S.D. (n = 3). *, *p *< 0.05 compared with CON, CON, control treated with vehicle. (C) Cells were transfected with NF-κB reporter gene with CYLD siRNA or empty vector. *S.p.*- or PLY-induced NF-κB promoter activity was measured as relative luciferase activity. (D) Cells were transfected either with CYLD siRNA or empty vector, and *S.p.*-induced TLR2 mRNA expression was measured 5 hours after treatment by Q-PCR analysis. (E) WT and CYLD KO mice were intratracheally inoculated with *S.p.*, and TLR2 mRNA expression was measured from the lungs of inoculated mice 6 hours after inoculation by Q-PCR analysis. Data are means ± S.D. (n = 3). *, *p *< 0.05 compared with CON, **, *p *< 0.05 compared with *S.p. *in MOCK (A-C) or in WT mice (D), CON, control inoculated with vehicle, MOCK, transfected with empty vector.

Bacteria modulates many innate immune responses via induction of negative regulatory molecules [15,17,18]. We previously reported that *S. pneumoniae *induces tumor suppressor CYLD, an important negative regulator of NF-κB signaling pathway, in severe *S. pneumoniae *infection [15]. NTHi induced CYLD expression, which in turn leads to negative regulation of NTHi-induced NF-κB-dependent inflammation [19]. CYLD expression is relatively low in lung but is highly induced under diseased condition [18]. To determine the role of CYLD in *S. pneumoniae*-induced TLR2 expression in the lung and middle ear, we first evaluated up-regulation of CYLD *in vitro*. As shown in Fig. 3A, *S. pneumoniae *induced CYLD expression not only in lung epithelia cell A549, but also in middle ear epithelial cell HMEEC-1. To further confirm whether *S. pneumoniae *induces CYLD both in the lung and middle ear *in vivo*, WT mice were intratracheally or transtympanically inoculated with *S. pneumoniae*, and CYLD mRNA expression was then measured in the lung and middle ear tissues of inoculated mice, respectively. As shown in Fig. 3B, *S. pneumoniae *induced CYLD expression in both lung and middle ear of the mice.

To further investigate the role of CYLD in *S. pneumoniae*-induced NF-κB activation, the effect of CYLD knockdown using CYLD siRNA was measured. As shown in Fig. 3C. *S. pneumoniae*- and PLY-induced NF-κB promoter activity was greatly enhanced by CYLD siRNA. To investigate whether CYLD acts as a negative regulator for *S. pneumoniae*-induced TLR2 expression, the effects of CYLD siRNA on *S. pneumoniae*-induced TLR2 mRNA expression were measured. As shown in Fig. 3D, knockdown of CYLD expression using CYLD siRNA greatly enhanced *S. pneumoniae*-induced TLR2 mRNA expression. To determine the *in vivo *role of CYLD on *S. pneumoniae*-induced TLR2 expression, we measured TLR2 mRNA expression in lung tissues of WT and CYLD KO mice after *S. pneumoniae *inoculation. Consistent with our *in vitro *findings, *S. pneumoniae*-induced TLR2 mRNA up-regulation was enhanced in CYLD KO mice (Fig. 3E). Together, our data indicate that CYLD acts as a negative regulator for *S. pneumoniae*-induced NF-κB activation and TLR2 up-regulation.

### *S. pneumoniae *synergizes with NTHi to induce inflammatory response via TLR2

**Figure 4:**
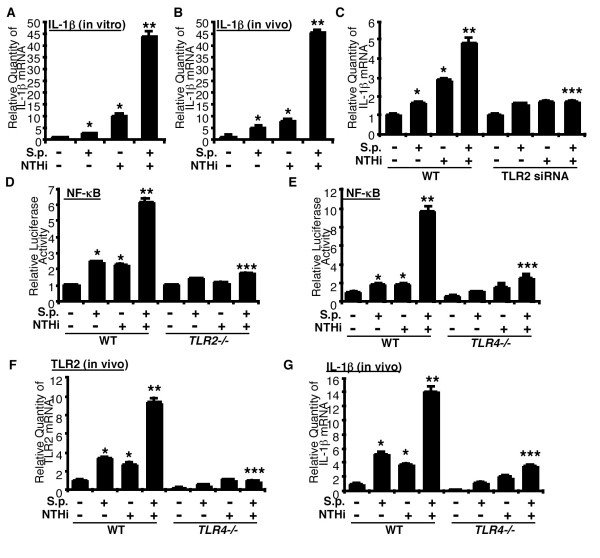
***S. pneumoniae *synergizes with NTHi to induce inflammatory response by up-regulating TLR2 expression**. (A) HeLa cells were treated with *S.p. *with or without NTHi, and IL-1β mRNA expression was measured 5 hours after treatment by Q-PCR analysis. (B) WT mice were intratracheally inoculated with *S.p. *with or without NTHi, and IL-1β mRNA expression was measured from the lungs of inoculated mice 6 hours after inoculation by Q-PCR analysis. (C) Cells were transfected with TLR2 siRNA or control siRNA, treated with *S.p. *with or without NTHi, and IL-1β mRNA expression was measured 5 hours after infection by Q-PCR analysis. (D) NF-κB luciferase activity following *S.p.*, NTHi, or *S.p. *with NTHi in MEF cells from WT and TLR2 KO mice. MEF cells from WT and TLR2 KO mice were transfected with NF-κB reporter gene, inoculated with *S.p.*, NTHi, or *S.p. *with NTHi, and relative luciferase activity was measured 5 hours after inoculation. (E) NF-κB luciferase activity following *S.p.*, NTHi, or *S.p. *with NTHi in MEF cells from WT and TLR4 KO mice. MEF cells from WT and TLR4 KO mice were transfected with NF-κB reporter gene, inoculated with *S.p.*, NTHi, or *S.p. *with NTHi, and relative luciferase activity was measured 5 hours after inoculation. (F & G) TLR2 and IL-1β up-regulation in the lungs of WT and TLR4 KO mice following *S.p.*, NTHi, or *S.p. *with NTHi *in vivo*. WT and TLR4 KO mice were intratracheally inoculated with *S.p.*, NTHi, or *S.p. *with NTHi, and TLR2 (F) and IL-1β (G) mRNA expression was measured from the lung of inoculated mice 6 hours after inoculation. Data are means ± SD (n = 3). *, *p *< 0.05 compared with control, **, *p *< 0.05 compared with NTHi treatment, ***, *p *< 0.05 compared with *S.p. *with NTHi treatment in MOCK or WT mice.

To determine whether *S. pneumoniae *synergizes with NTHi to induce inflammatory response, we investigated the effect of *S. pneumoniae *and NTHi on the expression of IL-1β, a key NF-κB-dependent pro-inflammatory cytokine in lung and middle ear. As shown in Fig. 4A, *S. pneumoniae *and NTHi synergistically enhanced expression of IL-1β ? mRNA in human lung epithelial cell. Moreover, *S. pneumoniae *and NTHi also synergistically induced expression of IL-1β mRNA in the lungs of mice (Fig. 4B). Next we investigated the role of TLR2 in synergistic induction of NF-κB-dependent inflammatory response by *S. pneumoniae *and NTHi. As shown in Fig. 4C, siRNA mediated knockdown of TLR2 markedly inhibited synergistic induction of IL-1β mRNA expression by *S. pneumoniae *and NTHi, thereby providing evidence for the involvement of TLR2 in this synergistic induction. This finding was further confirmed in the cells from TLR2 KO mice *in vitro *(Fig. 4D). Having shown that *S. pneumoniae*-induced TLR2 up-regulation is inhibited by TLR4-deficiency, and TLR2 up-regulation is closely associated with synergistic response by *S. pneumoniae *with NTHi, we determined if TLR4-deficiency affects synergistic activation of NF-κB and subsequent gene expression by *S. pneumoniae *with NTHi. We first measured NF-κB activation and mRNA expressions of TLR2 and IL-1β following co-treatment with *S. pneumoniae *and NTHi in cells (Fig. 4E) and lung tissues (Fig. 4F &4G) of WT and TLR4 KO mice. As shown in Fig. 4E, NF-κB activation by *S. pneumoniae *with or without NTHi was significantly decreased in the cells from TLR4 KO mice compared with those in cells from WT mice (Fig. 4E). In addition, mRNA expressions of TLR2 and IL-1β by *S. pneumoniae *with or without NTHi in the lung tissues of TLR4 KO mice were also significantly decreased compared with those in WT mice (Fig. 4F &4G). Taken together, these findings suggest that *S. pneumoniae *synergizes with NTHi to induce NF-κB activation and subsequent gene expression via TLR2 up-regulation.

## Discussion

There is a growing body of evidence showing that a portion of patients diagnosed with OM or COPD have mixed infections of NTHi and *S. pneumoniae *[4,5]. Under *in vivo *situations such as polymicrobial infections, mucosal epithelial surfaces are simultaneously exposed to a variety of bacteria and bacterial components, and one microbe synergizes with another one to induce the inflammatory response by activating intracellular signaling pathway [6,7]. Among many other innate immune components, TLRs are critical for initiating and mediating immune response. Thus, regulation of TLRs expression plays an important role in modulating host response to microbes. However, little is known about the molecular mechanisms underlying regulation of TLRs expression in mixed infection.

As we previously reported, TLR2 expression is relatively low in unstimulated epithelial cells but is markedly up-regulated in response to invading microbes [10,11]. However, the link between TLR2 up-regulation and mixed infection in otitis media and pneumonia still remains unknown. From what we have shown in the present data, it is evident that TLR2 expression is up-regulated by *S. pneumoniae *via a TLR4-MyD88-NF-κB-dependent signaling pathway through its well-known virulence factor PLY. We further show that *S. pneumoniae *and NTHi synergize with each to induce NF-κB activation and IL-1β expression dependently of TLR2 up-regulation. In the present study, we provided evidence for the first time that *S. pneumoniae *synergizes with NTHi to induce NF-κB-dependent inflammatory response likely via TLR2 up-regulation in lung and middle ear epithelia cells, thereby bringing new insight into our understanding of the complex regulatory mechanisms underlying inflammation in polymicrobial infections.

In addition to TLR2 and TLR4, TLR1, 6, and 9 have also been shown to be involved in *S. pneumoniae *infection [20-22]. Therefore we evaluated lung epithelial cell expression of 10 TLRs (TLR1-10) and CD14, MyD88, MD1 and 2 following *S. pneumoniae *treatment in A549 cells. In untreated cells, the expression level of TLR1, 3, 4, 5, 6 and 9 was relatively high compared to those of TLR2, 7, and 8. Interestingly, the expression of TLR2, 7 and 8 at the mRNA level was highly up-regulated following *S. pneumoniae *treatment (Data not shown), implying a possible important role for TLR2 up-regulation in bacterial infections. Indeed, the data we presented in this study demonstrated that the synergistic activation of NF-κB and inflammation by *S. pneumoniae *and NTHi is dependent on TLR2 up-regulation.

Although there is strong evidence to support that TLR4 is a critical receptor for recognizing pneumolysin and pneumolysin is a major virulence factor of pneumococcal infection, the role of TLR4 in pneumococcal infection is not fully understood [23-26]. It remains somewhat controversial whether TLR4-deficiency confers susceptibility or tolerance to pneumococcal infection *in vivo *[23,24,27,28]. Controversial phenotype of TLR4-deficiency mice in *S. pneumoniae *infection may be partly due to the strain differences of the mice used in each experiment, e.g. using C3H/HeJ, BALB/c and C57BL/6 as background strains. It is well known that different mouse strains exhibit a very different susceptibility to pneumococcal infection [28-30]. BALB/c mice, which were used as a background strain of TLR4 KO mice in our current experiment, were known as resistant strain in pneumococcal infection compared to other strains [30]. TLR4-deficiency in this strain thus may have no significant effect on pneumococcal clearance.

Another interesting finding in this study is the experimental evidence for the negative regulation of *S. pneumoniae*-induced TLR2 up-regulation by tumor suppressor CYLD. CYLD was previously identified as an important regulator not only in tumor development [31,32] but also in adaptive immune response [33,34]. Recent evidence demonstrated that CYLD also serves as a key regulator for innate immune response via negative-crosstalk with MAPKs and NF-κB [15,17,19,32]. Moreover we recently identified an important role for CYLD in modulating host antiviral response by regulating TLR7 expression in mixed infection of bacteria and virus [35]. However, still unknown is how CYLD regulates inflammatory response in mixed bacterial infection.

## Conclusion

In the present study we showed for the first time that *S. pneumoniae *synergizes with NTHi to induce NF-κB activation and the subsequent inflammatory response via up-regulation of TLR2. Moreover, we showed that *S. pneumoniae*-induced TLR2 up-regulation is mediated by TLR4-MyD88-IKKα-IκBα signaling pathway. Interestingly, tumor suppressor CYLD acts as a negative regulator for the synergistic induction of inflammation by *S. pneumoniae *and NTHi, thereby implying a critical role for CYLD in preventing overactive inflammatory response in mixed infections. This study may bring new insights into the molecular mechanisms underlying polymicrobial infections, especially mixed infection of gram-positive and negative bacteria.

## Methods

### Cell cultures

Human alveolar epithelial cell line A549, human middle ear epithelia cell line HMEEC-1 derived by human papillomavirus immortalization of primary human middle ear epithelial cell, and human cervix epithelia cell line HeLa were maintained as described previously [6,9-12]. Mouse embryonic fibroblasts (MEFs) were isolated from E13 embryos of WT, TLR2 KO and TLR4 KO mice and cultured in DMEM.

### Bacteria and pneumolysin

Clinical isolates of *S. pneumoniae *WT D39, and D39 isogenic PLY-deficient mutant PLN were used in both *in vitro *and *in vivo *experiments [36]. *S. pneumoniae *was grown on chocolate agar plates and in Todd-Hewitt broth supplemented with 0.5% yeast extract (THY) at 37°C in a humidified 5% CO_2 _water-jacketed incubator without shaking. Mid-log phase bacterial culture was used in the experiments. *S. pneumoniae *was inoculated at the concentration of 1 × 10^6 ^or 1 × 10^7 ^colony forming unit (CFU) in both *in vitro *and *in vivo *experiments unless otherwise specifically indicated in the figures and figure legends. Native PLY was described previously [25] and treated/inoculated at the concentration of 100 ng/ml. NTHi strain 12, a clinical isolate, was used in this study. NTHi was inoculated at the concentration of 1 × 10^7 ^CFU in both *in vitro *and *in vivo *experiments. The preparation of bacterial lysate was described previously [10,14].

### Animal and animal experiments

C57BL/6 and BALB/c mice were purchased from Charles River. TLR4 KO mice were purchased from Jackson Lab., and TLR2 and MyD88 KO mice were kindly provided by Dr. S. Akira. CYLD KO mice were reported previously [15].

For the *S. pneumoniae *or PLY-induced pneumonia model in mice, animals were intratracheally inoculated with 1 × 10^7 ^CFU of *S. pneumoniae *or 100 ng of PLY in 50 μl of saline. At the time point indicated in the figure legends, mice were sacrificed by overdose injection of sodium pentobarbital, and mRNA expressions of TLR2, CYLD, and IL-1β were measured in the lung tissues of control and *S. pneumoniae *or PLY-inoculated mice as described below. For the *S. pneumoniae *or PLY-induced OM model in mice, animals were inoculated with *S. pneumoniae *or PLY in 10 μl of saline via transtympanic inoculation. Nine hours after inoculation, animals were sacrificed by overdose injection of sodium pentobarbital, and middle ears were dissected from the skull for extraction of mRNA for measuring TLR2 and CYLD expression. For the control inoculation in both pneumonia and OM model, 50 μl or 10 μl of saline was inoculated, respectively. All animal experiments were approved by the Institutional Animal Care and Use Committee at University of Rochester.

### Transfection and small interfering RNA (siRNA)

The expression plasmids including TLR4 dominant-negative (DN) mutant, IκBα DN (S32/36A), IKKα DN (K44M), and NF-κB and TLR2 luciferase reporters were described previously [6,9-12]. RNA-mediated interference for down-regulating CYLD, IKKα, and TLR2 expression was conducted using small interfering CYLD siRNA (pSuper-CYLD), IKKαsiRNA (Dharmacon), and TLR2 siRNA (Dharmacon) as described previously [17]. All transient transfections of plasmids were carried out using TransIT-LT1 (Mirus), and RNA-mediated interferences were carried out using Lipofectamine™ 2000 (Invitrogen) following the manufacturer's instructions.

### Real-time quantitative PCR (Q-PCR) analysis

Q-PCR analysis of TLR2, CYLD, and IL-1β was conducted as follows. Total RNA was isolated from cells and lung and middle ear tissues of mice using TRIzol (Invitrogen) following the manufacturer's instruction. RT reaction was conducted using TaqMan reverse transcription reagents (ABI) following the manufacturer's instruction. PCR amplification was performed with TaqMan universal master mix (ABI) for TLR2 and IL-1β and SYBR green universal master mix (ABI) for CYLD. Predeveloped human and mouse TLR2 and IL-1β Q-PCR primers were purchased from ABI, and primer sequences for human and mouse CYLD are as follows: human CYLD sense, 5'-ttc agc ctg ttt aaa aac aga aa-3'; human CYLD antisense, 5'-tcc cca gga cct gcg taa t-3'; mouse CYLD sense, 5'-ctc agc cta ttt aga aac aga ct-3'; mouse CYLD antisense, 5'-tct cct ggg cct gca aat-3'. Reactions were amplified and quantified using an ABI 7500 sequence detector and manufacturer's software (ABI). The relative quantity of TLR2, CYLD, and IL-1β mRNA was obtained using the comparative CT method and was normalized using predeveloped TaqMan assay reagent human cyclophilin and mouse GAPDH as an endogenous control for human mRNA and mouse mRNA, respectively.

### Western Blotting analysis

Antibody against TLR2 was purchased from eBioscience, and Western blot analysis of TLR2 was conducted as follows. WT mice were first intratracheally inoculated with 1 × 10^7 ^CFU of *S. pneumoniae *and total protein was then extracted from the lung tissues of saline- or *S. pneumoniae*-inoculated mice 6 hours after inoculation. Western blot analysis was performed as described previously [10,14,37].

### Statistical analysis

Statistical analysis was performed with student t-test. *P *values of less than 0.05 were considered statistically significant.

## Abbreviations

*S. pneumoniae*: *Streptococcus pneumoniae*; NTHi: nontypeable *Haemophilus influenza*; PLY: pneumolysin; TLR: Toll-like receptor; CYLD: Cylindromatosis; NF-κB: nuclear factor-kappa B; WT: wild-type; KO: knock-out; Q-PCR: real-time quantitative RT-PCR.

## Authors' contributions

The authors contributed to the work as following: JHL designed and performed experiments, collected and analyzed data, and prepared manuscript; UH and AS designed research and performed experiment; CHW and HX analyzed the data and contributed to the preparation of the manuscript; SKW performed experiments; JDL conceived study, designed research, analyzed data, and wrote the manuscript. All authors read and approved the final manuscript.
